# Reliability of Myotonometric Measurements and Their Associations with Motor Performance in Parkinson’s Disease

**DOI:** 10.3390/s26206410

**Published:** 2026-10-10

**Authors:** Lucía Latorre-Tomillo, Eduardo Villamil-Cabello, Noa Fogelson, Miguel Ángel Fernández-del-Olmo

**Affiliations:** Center for Research in Sport Sciences, Rey Juan Carlos University, Camino del Molino 5, 28942 Fuenlabrada, Madrid, Spain; l.latorre.2025@alumnos.urjc.es (L.L.-T.); eduardo.villamil@urjc.es (E.V.-C.); noa.fogelson@urjc.es (N.F.)

**Keywords:** Parkinson’s disease, myotonometry, reliability, gait

## Abstract

**Highlights:**

- Under the standardized same-session, same-rater protocol, MyotonPRO-derived muscle mechanical parameters showed good-to-excellent within-session reliability point estimates in people with Parkinson’s disease.
- Several moderate nominal associations were observed with preferred gait and manual dexterity, but none remained significant after false discovery rate correction and should be considered hypothesis-generating.

**Abstract:**

Myotonometry provides a non-invasive assessment of resting muscle mechanical properties, but its reliability and functional relevance in Parkinson’s disease (PD) remain insufficiently established. This study examined the within-session intra-rater reliability of MyotonPRO measurements and explored their associations with clinical motor severity, gait, functional mobility, and manual dexterity in 24 people with PD assessed in the ON-medication state. Frequency, stiffness, logarithmic decrement, relaxation time, and creep were measured bilaterally in the biceps brachii, triceps brachii, tibialis anterior, and medial gastrocnemius. Reliability between two sets of five impulses was assessed using ICC(3,2), and associations were examined using Spearman correlations with Benjamini–Hochberg false discovery rate correction. Under the standardized same-session, same-rater protocol, all parameters showed good-to-excellent reliability point estimates (ICC = 0.823–0.988), although the precision of some estimates was limited by relatively wide confidence intervals. No significant associations were found with MDS-UPDRS III scores, Hoehn and Yahr stage, or Timed Up and Go performance. Several moderate nominal correlations were observed between lower-limb myotonometric parameters and preferred gait performance, and between triceps brachii parameters and Grooved Pegboard completion time (|r_s_| = 0.410–0.488); however, none remained significant after false discovery rate correction. Thus, the reliability findings apply to the present standardized protocol, whereas the associations with motor performance remain exploratory and hypothesis-generating.

## 1. Introduction

Parkinson’s disease (PD) is a progressive neurodegenerative disorder characterized clinically by bradykinesia in combination with rigidity, resting tremor, or postural instability, as well as a broad range of non-motor manifestations [1]. Motor impairments contribute substantially to limitations in walking, upper-limb function, independence, and quality of life. Although these impairments originate predominantly from dysfunction of central neural circuits, increasing evidence indicates that peripheral neuromuscular and musculoskeletal factors may also contribute to the motor phenotype of PD [2].

Rigidity is one of the cardinal motor signs of PD and is clinically defined as increased resistance to externally imposed passive movement. However, the resistance detected during passive joint mobilization is a complex phenomenon that may reflect both neural and non-neural components. Neural mechanisms include abnormal muscle activation and altered responses to muscle stretch, whereas non-neural components arise from the intrinsic mechanical properties of muscles, tendons, connective tissues, and other periarticular structures [2,3]. Consequently, clinical rigidity should not be considered equivalent to muscle stiffness or resting muscle mechanical tone, as these constructs represent related but distinct aspects of motor and tissue behavior.

In clinical practice, motor impairment and rigidity are commonly assessed using the Movement Disorder Society–Unified Parkinson’s Disease Rating Scale Part III (MDS-UPDRS III). This scale is essential for the clinical characterization and monitoring of PD, but the assessment of rigidity depends partly on the examiner’s perception of resistance during passive movement. In addition, global clinical scales do not directly quantify the mechanical or viscoelastic properties of individual muscles [3]. Quantitative assessment of these properties may therefore provide complementary information on peripheral tissue characteristics that cannot be derived directly from conventional neurological examination.

Myotonometry comprises a group of non-invasive methods designed to quantify the mechanical behavior of superficial biological tissues in response to a controlled external mechanical stimulus. Depending on the measurement system, myotonometric assessment may provide information related to resting mechanical tone, resistance to deformation, elasticity, relaxation behavior, and viscoelasticity. These parameters characterize the mechanical state of the tissue under standardized conditions and should not be interpreted as direct measures of neural rigidity, voluntary force production, or electrically evoked contractile performance [4].

In the present study, myotonometric measurements were obtained using MyotonPRO (Myoton AS, Tallinn, Estonia), a handheld impulse-based device that applies a brief mechanical stimulus to the skin surface and records the resulting damped oscillation of the underlying tissue. From this response, the device derives oscillation frequency, dynamic stiffness, logarithmic decrement, mechanical stress-relaxation time, and creep-related measures. Oscillation frequency is commonly interpreted as an indicator of resting mechanical tone, dynamic stiffness represents resistance to an externally applied deformation, and logarithmic decrement reflects the dissipation of mechanical energy and is inversely related to tissue elasticity. Relaxation time and creep-related measures provide additional information about time-dependent viscoelastic behavior [4]. Although the reported variables capture complementary dimensions of tissue mechanics, their values are influenced by methodological factors such as the device used, anatomical site, participant positioning, muscle relaxation, probe placement, and measurement protocol. Studies in healthy participants and clinical populations, including people with Parkinson’s disease, have generally reported good-to-excellent reliability for myotonometric measurements [4,5].

However, reliability must be demonstrated under the specific conditions in which the technique is applied. This is especially relevant in Parkinson’s disease, where tremor, background muscle activity, motor fluctuations, difficulty maintaining complete relaxation, and small changes in positioning may affect repeated measurements. Evidence in this population remains limited, although previous work has shown that MyotonPRO assessment is feasible and can provide reliable within-session measurements [5].

Beyond reliability, the clinical relevance of myotonometry depends on whether resting muscle mechanical properties relate to meaningful motor outcomes. In Parkinson’s disease, gait impairment, functional mobility, and manual dexterity reflect complex interactions among central motor, biomechanical, and cognitive processes [6,7,8]. Lower-limb muscles such as the tibialis anterior and gastrocnemius contribute to foot clearance, ankle control, and propulsion, while upper-limb muscles support stabilization and movement control during manual tasks. However, the extent to which resting mechanical properties of these muscles are associated with gait, Timed Up and Go performance, or Grooved Pegboard dexterity remains unclear.

The lower-limb muscles were selected because the tibialis anterior and medial gastrocnemius contribute directly to ankle control, foot clearance, and propulsion during walking. The biceps brachii and triceps brachii were selected as superficial upper-limb muscles that can be assessed reproducibly with MyotonPRO and that contribute to proximal positioning and stabilization during upper-limb tasks, although they are not the primary muscles responsible for fine finger dexterity. Thus, the selected muscles provided standardized, accessible sites in both upper and lower limbs while allowing exploratory examination of relationships with clinically relevant motor outcomes.

Most previous studies examining muscle mechanics in PD have focused on comparisons with neurologically healthy individuals, the objective characterization of rigidity, or changes related to dopaminergic medication and deep brain stimulation [2,3,9]. Comparatively less attention has been given to the relationship between myotonometrically assessed muscle properties and instrumented gait outcomes, global functional mobility, and manual dexterity within a PD population. Studying these relationships may help determine whether myotonometry provides information that complements established clinical assessments.

Therefore, the aims of this cross-sectional study were: (1) to determine the intra-rater reliability of myotonometric measurements obtained with MyotonPRO from selected upper- and lower-limb muscles in people with PD; and (2) to explore the associations between resting muscle mechanical properties and clinical motor severity, spatiotemporal gait performance, global functional mobility, and manual dexterity. We hypothesized that most myotonometric parameters would demonstrate acceptable within-session reliability. We also hypothesized that selected muscle mechanical properties would be associated with functionally related motor outcomes.

## 2. Materials and Methods

### 2.1. Study Design

This study was a cross-sectional analysis of data collected as part of a randomized clinical trial registered at ClinicalTrials.gov (NCT07524400), which investigated the effects of transcranial direct current stimulation (tDCS) combined with exercise in people with Parkinson’s disease. The present analysis included a subset of 24 participants from the total sample of 100 participants enrolled in the parent trial. The myotonometric reliability and association analyses reported here were conducted as exploratory analyses.

All assessments were conducted at the Motor Control Laboratory of Rey Juan Carlos University, Madrid, Spain.

### 2.2. Participants

Twenty-four individuals diagnosed with idiopathic PD were included in the present analysis. Participants were recruited from local Parkinson’s associations. The diagnosis of PD had been established by a neurologist according to accepted clinical diagnostic criteria. Mean disease duration was 8.34 ± 5.14 years. Cognitive status was assessed using the Mini-Mental State Examination (MMSE), with a mean score of 25.30 ± 3.48.

The inclusion criteria were: (1) a clinical diagnosis of PD; (2) age ≥ 18 years; (3) ability to walk independently for at least 10 m; and (4) ability to understand and complete the assessment procedures. The exclusion criteria were: (1) surgery involving the upper or lower limbs during the previous six months; (2) acute musculoskeletal pain that limited performance of the functional assessments; and (3) inability to maintain the standardized positions required for the myotonometric measurements.

The MMSE was used for descriptive purposes only, and no cognitive cut-off score was applied as an exclusion criterion. Participants were required to understand the study instructions and complete the assessment procedures. The sample was relatively homogeneous in terms of clinical status: most participants were independently ambulatory, were concentrated within Hoehn and Yahr stages 2–2.5, and presented an akinetic-rigid motor phenotype.

The sample size was estimated a priori using G*Power software (version 3.1.9.7, Heinrich Heine University Düsseldorf, Düsseldorf, Germany). Assuming a two-tailed correlation, an alpha level of 0.05, statistical power of 0.80, and an expected correlation coefficient of 0.55, the estimated minimum sample size was 23 participants. Twenty-four participants were included in the final analysis. The a priori sample-size calculation was based on the association analyses and was not derived from an expected ICC or a target confidence-interval width for the reliability analysis.

### 2.3. Experimental Procedure

Participants attended the laboratory on two occasions. The first session was devoted to familiarization with the motor tests to minimize potential learning effects. All study assessments were conducted during the second session one week later, including clinical scales, gait and functional mobility tests, manual dexterity assessment, and myotonometric measurements.

During the second laboratory session, the order of the clinical assessment, gait evaluation, Timed Up and Go test, Grooved Pegboard Test, and myotonometric measurements was randomized separately for each participant to minimize systematic order effects.

Both sessions were scheduled at a time of day when participants usually experienced a good response to their antiparkinsonian medication. Upon arrival, participants were asked to confirm that they perceived themselves to be in their usual ON-medication state, and testing began only after this confirmation. No additional standardized clinical confirmation of ON status was performed. Detailed information regarding antiparkinsonian medication class, regimen, levodopa equivalent daily dose, and medication stability was not systematically recorded. The interval since the last antiparkinsonian medication dose was not standardized across participants. Participants maintained their usual medication regimen, and no medication withdrawal was required. Rest periods were provided before and between assessments when necessary to minimize fatigue. Myotonometric measurements were performed by the same trained evaluator.

The clinically more affected side was determined by comparing the summed scores of the lateralized MDS-UPDRS Part III items corresponding to the upper and lower limbs on the right and left sides. The side with the higher total score was classified as the more affected side. This classification was cross-checked against the participant’s self-reported more affected side, and no discrepancies were observed. No ties occurred in the study sample. This classification was subsequently used to distinguish the more affected and less affected sides in all analyses.

### 2.4. Clinical Assessment

Motor symptom severity was assessed using Part III of the Movement Disorder Society–Unified Parkinson’s Disease Rating Scale (MDS-UPDRS III). This clinician-administered examination evaluates motor manifestations including bradykinesia, rigidity, tremor, lower-limb agility, rising from a chair, posture, gait, and postural stability. Higher scores indicate greater motor impairment.

Disease stage was classified using the Hoehn and Yahr scale, which provides an ordinal description of disease progression based on the distribution of motor involvement and the presence of postural instability.

Both scales were administered by an evaluator trained in their application. The predominant motor phenotype was also recorded descriptively as akinetic-rigid or tremor-dominant. Because only four participants were classified as tremor-dominant, motor phenotype was not included in the inferential analyses.

### 2.5. Motor Performance Assessments

#### 2.5.1. Spatiotemporal Gait Assessment

Spatiotemporal gait parameters were recorded using the OptoGait optical detection system (Microgate, Bolzano, Italy). The system consists of parallel transmitting and receiving bars containing infrared light-emitting diodes. Interruptions of the infrared signals produced by foot contact allow the calculation of temporal and spatial gait parameters. OptoGait has previously demonstrated concurrent validity and test–retest reliability for the assessment of spatiotemporal gait variables [10].

Participants walked along an 8 m pathway using repeated outward and return trajectories for approximately 1 min. The OptoGait bars were positioned in parallel along the central 5 m of the pathway. Consequently, the initial acceleration, turning, and final deceleration phases were not included in the recorded section, allowing the analysis to focus predominantly on steady-state walking. This protocol was consistent with procedures previously used by our research group [11,12].

Two gait conditions were evaluated:

1. Preferred-speed walking: participants were instructed to begin walking when ready and to walk at their usual comfortable speed.
2. Maximal-speed walking: participants followed the same pathway but were instructed to walk as fast as safely possible without running.

The variables extracted under each condition were gait speed (m/s), step length (m), and cadence (steps/min). The mean value recorded across the valid passes within each 1 min condition was used for statistical analysis.

#### 2.5.2. Timed Up and Go Test

Functional mobility was evaluated using the Timed Up and Go test [7,13]. The test incorporates a sequence of locomotor actions involving sit-to-stand transfer, gait initiation, straight walking, turning, returning to the chair, and sitting down.

Participants began seated with their back supported against the chair. At the start signal, they were instructed to stand without using their upper limbs, walk 3 m at a comfortable and safe speed, turn 180°, return to the chair, and sit down. Timing began when the participant’s back lost contact with the chair and ended when the back regained contact after sitting.

Three trials were performed. Completion time was recorded using a commercially available digital stopwatch (Casio, Tokyo, Japan), and the mean time across the three trials was used for statistical analysis.

#### 2.5.3. Grooved Pegboard Test

Manual dexterity was assessed using the Grooved Pegboard Test (Model 32025; Lafayette Instrument Company, Lafayette, IN, USA). The apparatus consists of a 5 × 5 matrix containing 25 keyhole-shaped slots with different orientations. Because each peg contains a ridge along one side, participants must identify the orientation of each slot, rotate the peg accordingly, and insert it into the corresponding hole.

The task requires fine finger control, visuomotor coordination, movement speed, and manipulation accuracy. Grooved Pegboard performance has previously been shown to index motor impairment in people with PD independently of tremor severity [8].

The test was performed using the hand corresponding to the clinically more affected side. Participants were instructed to insert all 25 pegs as quickly and accurately as possible. Completion time was measured in seconds, and the test was discontinued after 300 s if the participant had not completed the task.

### 2.6. Myotonometric Assessment

Resting muscle mechanical properties were assessed bilaterally using a MyotonPRO device (Myoton AS, Tallinn, Estonia). The device applies a brief mechanical impulse perpendicular to the skin surface and records the damped oscillatory response of the underlying tissue.

The following device-derived parameters were recorded:

- Oscillation frequency (Hz): the natural frequency of tissue oscillation, commonly interpreted as an indicator of resting mechanical tone;
- Dynamic stiffness (N/m): the resistance of the tissue to deformation produced by the mechanical impulse;
- Logarithmic decrement: the dissipation of mechanical energy between successive oscillations, with higher values indicating lower elasticity;
- Mechanical stress-relaxation time (ms): the time required for the tissue to recover from deformation;
- Creep or Deborah number: a dimensionless parameter describing the relationship between relaxation and deformation time and, therefore, the time-dependent viscoelastic behavior of the tissue.

#### 2.6.1. Muscles and Measurement Sites

Measurements were obtained bilaterally from the biceps brachii, triceps brachii, tibialis anterior, and medial gastrocnemius. Standardized anatomical locations and participant positions were selected on the basis of previously published myotonometric protocols [5,14,15]:

Biceps brachii. Participants were seated with the elbow extended, the forearm supinated, and the arm fully supported and relaxed. A towel was placed beneath the elbow when necessary to facilitate muscle relaxation. The measurement site was located over the muscle belly at approximately 75% of the distance between the acromion and the cubital fossa.

Triceps brachii. Measurements were obtained over the long head of the triceps brachii at the midpoint of the distance between the posterior acromion and the olecranon. All participants were assessed in the prone position with the arm relaxed alongside the body. A supported seated position had been predefined as an alternative if prone positioning was not feasible, based on previously published procedures, but this alternative was not required for any participant.

Tibialis anterior. Participants were positioned supine with the lower limbs relaxed and the ankle in a neutral position. The measurement site was located over the tibialis anterior muscle belly in the proximal third of the lower leg. The site was identified by palpation during a brief voluntary dorsiflexion, after which the participant was instructed to relax completely before the measurement was obtained.

Medial gastrocnemius. Participants were positioned prone with the feet extending beyond the end of the examination table to avoid external support of the ankle. The measurement point was located over the medial gastrocnemius at approximately 33% of the distance between the popliteal crease and the calcaneus.

All measurement points were marked with a dermographic pen to ensure that repeated measurements were obtained from the same anatomical location. The probe was positioned perpendicular to the skin surface, and the underlying muscle was required to remain completely relaxed.

Before each measurement, the tested limb was fully supported, and participants were instructed to remain still and completely relax the target muscle. The evaluator visually inspected the limb and muscle region for overt voluntary contraction or movement before applying the probe. If movement, visible contraction, or difficulty maintaining relaxation was observed, the measurement was delayed and repeated after repositioning and renewed relaxation instructions. Muscle relaxation was not objectively verified using electromyography.

#### 2.6.2. Measurement Protocol

The device was operated in Pattern mode. Each measurement set consisted of five consecutive mechanical impulses delivered automatically at the same anatomical location. Two measurement sets were obtained for every muscle and side.

This procedure was based on previous reliability research in people with PD in which repeated sets of five impulses were used to assess muscle mechanical properties [5]. Other investigations have similarly used repeated impulse sets to improve measurement stability and quantify within-session reliability [4,16].

For each measurement set, the device automatically calculated the coefficient of variation (CV) across the five impulses. In accordance with the manufacturer’s recommended procedure, a CV threshold of <3% was used as an a priori quality-control criterion. Measurements exceeding this threshold were repeated after checking participant relaxation, probe placement, and limb position. This criterion has also been used in previous MyotonPRO reliability protocols [17]. A CV >3% occurred on approximately seven occasions during the study. In all cases, an acceptable measurement was obtained within a maximum of three attempts, and no measurement was excluded because of failure to meet the <3% criterion.

All measurements were obtained by the same evaluator. The two accepted measurement sets were retained separately for the intra-rater reliability analysis. The mean of both sets was used in the analyses examining associations with clinical and functional outcomes.

### 2.7. Statistical Analysis

Statistical analyses were performed using JASP software (version 0.98, JASP Team, Amsterdam, The Netherlands). Continuous variables are presented as mean and standard deviation, whereas sex, motor phenotype and Hoehn and Yahr stage are reported as frequencies and percentages. The distribution of continuous variables was examined using the Shapiro–Wilk test. All 24 participants had complete data for the variables included in the present analyses; therefore, no missing-data procedure was required.

#### 2.7.1. Reliability Analysis

Within-session intra-rater reliability was evaluated separately for each muscle, side, and myotonometric parameter. Each measurement set consisted of five consecutive mechanical impulses. The arithmetic mean of the five impulses was calculated for each set, resulting in one mean value for the first measurement set and one mean value for the second measurement set.

Because the inferential analyses used the average of the two accepted five-impulse measurement sets, an average-measures intraclass correlation coefficient was selected. ICC(3,2) was calculated using a two-way mixed-effects model with participants treated as random and the repeated measurement condition treated as fixed, because the same examiner and standardized protocol were applied to all participants. A consistency definition was used to quantify the stability of participants’ relative ranking across repeated measurements obtained under identical standardized conditions. Accordingly, ICC(3,2) represents the reliability of the average of two set means rather than the reliability of a single impulse or a single measurement set. ICC estimates were reported together with their 95% confidence intervals.

ICC values were interpreted according to Koo and Li [18]: values below 0.50 indicated poor reliability, values between 0.50 and 0.75 indicated moderate reliability, values between 0.75 and 0.90 indicated good reliability, and values above 0.90 indicated excellent reliability.

#### 2.7.2. Association Analyses

Spearman’s rank correlation coefficient (r_s_) was used because several variables did not meet the assumptions for Pearson correlation, including approximate normality and linearity, and because Hoehn and Yahr stage is an ordinal variable.

The association analyses examined relationships between:

1. myotonometric parameters and MDS-UPDRS III score;
2. myotonometric parameters and Hoehn and Yahr stage;
3. tibialis anterior and medial gastrocnemius properties and gait speed, step length, and cadence during preferred- and maximal-speed walking;
4. biceps brachii and triceps brachii properties from the clinically more affected side and Grooved Pegboard completion time;
5. tibialis anterior and medial gastrocnemius properties and mean TUG completion time.

All tests were two-sided, and the nominal significance level was set at *p* < 0.05. To account for multiple testing, the Benjamini–Hochberg false discovery rate (FDR) procedure was applied separately within four outcome-based families of correlation tests: clinical severity (80 tests), gait performance (120 tests), manual dexterity (10 tests), and functional mobility (20 tests). The gait family included preferred- and maximal-speed walking but excluded dual-task conditions. FDR-adjusted *p*-values were calculated using all tests within each respective family, irrespective of whether the unadjusted *p*-value was below 0.05. Both unadjusted and FDR-adjusted *p*-values are reported for associations reaching nominal significance (unadjusted *p* < 0.05). Because multiple parameters, muscles, sides, and functional outcomes were examined in a relatively small sample, the association analyses were considered exploratory and hypothesis-generating.

### 2.8. Ethical Considerations

The study was conducted in accordance with the Declaration of Helsinki and was approved by the Research Ethics Committee of Universidad Rey Juan Carlos (reference 201120245922024).

All participants received oral and written information about the procedures and provided written informed consent before participation.

## 3. Results

### 3.1. Participant Characteristics

Twenty-four participants with Parkinson’s disease were included in the analysis, and all 24 completed the assessments included in the present analyses. The sample comprised 14 men and 10 women, with a mean age of 66.79 ± 9.15 years. Mean disease duration was 8.34 ± 5.14 years, and mean cognitive status assessed using the MMSE was 25.30 ± 3.48 points. Mean motor severity, assessed using the MDS-UPDRS III, was 32.96 ± 13.22 points.

Most participants were classified in Hoehn and Yahr stages 2 and 2.5. Specifically, one participant was classified as stage 1, four as stage 1.5, nine as stage 2, seven as stage 2.5, and three as stage 3. Twenty participants were classified as having an akinetic-rigid phenotype and four as having a tremor-dominant phenotype.

Mean preferred gait speed was 1.11 ± 0.18 m/s, mean step length was 0.69 ± 0.07 m, and mean cadence was 112.79 ± 8.54 steps/min. Mean Timed Up and Go completion time was 8.34 ± 2.02 s. Mean Grooved Pegboard completion time with the more affected hand was 159.79 ± 77.08 s. Participant characteristics are presented in Table 1.

### 3.2. Intra-Rater Reliability of Myotonometric Measurements

The intra-rater reliability results are presented in Table 2. Reliability was good to excellent for all myotonometric parameters across the muscles and sides assessed. ICC values ranged from 0.823 to 0.988.

Oscillation frequency showed good-to-excellent reliability, with ICC values ranging from 0.868 to 0.988. Dynamic stiffness also demonstrated good-to-excellent reliability, with values between 0.823 and 0.985. Logarithmic decrement showed consistently excellent reliability across all measurement sites, with ICC values ranging from 0.934 to 0.988. Similarly, relaxation time and creep demonstrated good-to-excellent reliability, with values ranging from 0.829 to 0.984 and from 0.850 to 0.984, respectively.

The highest reliability estimates were generally observed for the tibialis anterior and medial gastrocnemius on the less affected side. The lowest ICC was obtained for biceps brachii stiffness on the less affected side. Although all point estimates fell within the good-to-excellent range, the precision of the estimates varied across muscles and parameters. Some 95% confidence intervals were relatively wide; for example, biceps brachii stiffness on the less affected side had an ICC of 0.823 with a 95% confidence interval of 0.590–0.923. The reliability findings should therefore be interpreted together with the uncertainty reflected by these intervals.

**Figure 1:**
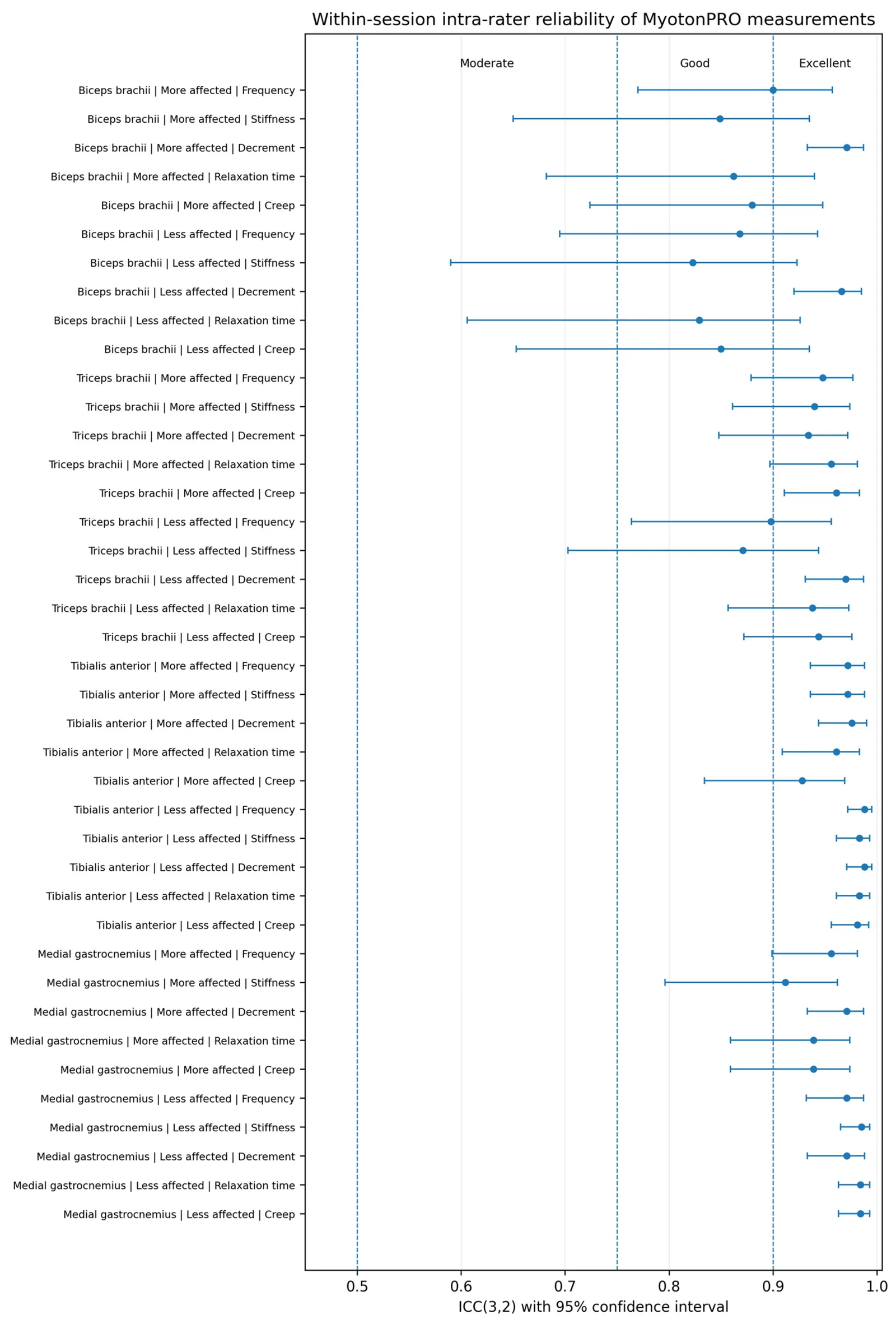
Forest plot of ICC(3,2) estimates and 95% confidence intervals for all myotonometric parameters, muscles, and sides. ICC(3,2) was calculated using a two-way mixed-effects model, consistency definition, and the average of two measurement-set means. Vertical reference lines indicate the commonly used thresholds for moderate (0.50), good (0.75), and excellent (0.90) reliability.

The 40 ICC(3,2) estimates and their 95% confidence intervals are additionally summarized graphically in Figure 1.

Values are ICC(3,2) estimates with 95% confidence intervals in parentheses. The ICC was calculated using a two-way mixed-effects model, consistency definition, and the mean of two measurement sets. According to Koo and Li [18], values below 0.50 indicate poor reliability, values from 0.50 to 0.75 indicate moderate reliability, values from 0.75 to 0.90 indicate good reliability, and values above 0.90 indicate excellent reliability. More affected and less affected sides were defined by comparing the summed lateralized MDS-UPDRS Part III upper- and lower-limb scores for the right and left sides; the side with the higher total was classified as more affected.

### 3.3. Associations with Clinical Motor Severity

No statistically significant associations were found between the myotonometric parameters and either MDS-UPDRS III scores or Hoehn and Yahr stage.

Across the four muscles, both sides, and the five myotonometric parameters, the Spearman correlation coefficients with MDS-UPDRS III were generally small. Similarly, no consistent pattern of association was observed between resting muscle mechanical properties and Hoehn and Yahr stage.

The complete correlation matrices for MDS-UPDRS III and Hoehn and Yahr stage are provided in Supplementary Tables S1 and S2, respectively.

### 3.4. Associations Between Lower-Limb Myotonometric Properties and Gait

No association between lower-limb myotonometric properties and gait performance remained statistically significant after correction for multiple comparisons.

Before FDR correction, several nominal associations were observed during preferred-speed walking. Higher oscillation frequency of the tibialis anterior on the more affected side was associated with shorter step length (*r*_s_ = −0.446, *p* = 0.029). In practical terms, participants with higher resting tibialis anterior oscillation frequency tended to show shorter preferred step length.

For the medial gastrocnemius, higher logarithmic decrement was associated with slower preferred gait speed on both the more affected side (*r*_s_ = −0.488, *p* = 0.016) and the less affected side (*r*_s_ = −0.410, *p* = 0.047). Longer relaxation time (*r*_s_ = −0.420, *p* = 0.041) and higher creep values (*r*_s_ = −0.465, *p* = 0.022) in the medial gastrocnemius on the more affected side were also nominally associated with slower preferred gait speed.

No nominally significant associations were observed between the myotonometric parameters and gait speed, step length, or cadence during maximal-speed walking. Table 3 summarizes only associations reaching nominal significance, whereas the complete correlation matrices for preferred- and maximal-speed walking are provided in Supplementary Tables S3 and S4, respectively.

### 3.5. Associations with Manual Dexterity

Two nominal associations were observed between the mechanical properties of the triceps brachii on the more affected side and Grooved Pegboard completion time.

Higher oscillation frequency was associated with longer completion time (*r*_s_ = 0.455, *p* = 0.026). In addition, lower logarithmic decrement was associated with longer completion time (*r*_s_ = −0.481, *p* = 0.017).

No corresponding associations were observed for dynamic stiffness, relaxation time, or creep, or for any of the biceps brachii parameters. Neither association remained statistically significant after FDR correction. Because the Grooved Pegboard primarily reflects distal fine-motor control whereas the myotonometric measurements were obtained from proximal upper-limb muscles, these nominal associations should be interpreted as indirect and exploratory. These results are summarized in Table 3, and the complete correlation matrix is provided in Supplementary Table S5.

### 3.6. Associations with Functional Mobility

No statistically significant associations were found between Timed Up and Go completion time and the myotonometric properties of the tibialis anterior or medial gastrocnemius on either side.

The correlation coefficients were small, ranging approximately from −0.11 to 0.23, and no consistent directional pattern was observed. The complete correlation matrix is provided in Supplementary Table S6.

A global heatmap summarizing the Spearman correlation coefficients across the association analyses is provided in Supplementary Figure S1.

## 4. Discussion

The present study examined the within-session intra-rater reliability of myotonometric measurements and explored their associations with clinical motor severity, gait performance, functional mobility, and manual dexterity in people with Parkinson’s disease. Under the standardized same-session, same-rater protocol used in this study, all myotonometric parameters showed good-to-excellent reliability point estimates across the muscles and sides assessed, although the precision of some estimates was limited by relatively wide confidence intervals. The first hypothesis was therefore supported. In contrast, the second hypothesis was not supported after correction for multiple comparisons: although several moderate associations reached nominal significance for preferred gait and manual dexterity, none remained significant after FDR correction. No significant associations were found with MDS-UPDRS III scores, Hoehn and Yahr stage, or Timed Up and Go performance. Accordingly, the association findings should be considered exploratory and hypothesis-generating rather than confirmatory.

### 4.1. Reliability of Myotonometric Measurements

All myotonometric parameters demonstrated good-to-excellent within-session intra-rater reliability, with ICC values ranging from 0.823 to 0.988. These findings are consistent with the systematic review by Lettner et al. [4], which concluded that MyotonPRO measurements generally show good reliability, although estimates vary according to the muscle, parameter, population, and assessment protocol. A broader review of myotonometry similarly identified measurement standardization, anatomical location, participant positioning, and tissue characteristics as relevant determinants of reliability [19].

Our findings also agree closely with those of Agoriwo et al. [5], who evaluated two sets of five MyotonPRO impulses in people with Parkinson’s disease and reported ICC values above 0.92 for the biceps brachii and tibialis anterior and above 0.86 for the flexor carpi radialis. The similarly high values obtained in the present study extend those observations to the triceps brachii and medial gastrocnemius and to all five parameters derived by the device. Agoriwo et al. also used a same-session, same-examiner design, making their protocol particularly comparable with ours. Taken together, the findings from both studies support the reproducibility of this specific within-session protocol; however, they should not be extrapolated directly to inter-rater or between-day reliability. Accordingly, the reliability findings apply specifically to the standardized same-session, same-rater protocol used in this study and should not be generalized to inter-rater or between-session reliability.

For comparison, Aird et al. [16] reported very high within-day reliability (ICC(3,1) > 0.90) and high between-day reliability (ICC(3,2) > 0.70) for MyotonPRO measurements of the rectus femoris in healthy older males. The present within-session ICC(3,2) estimates (0.823–0.988) are broadly consistent with the high reliability reported in healthy cohorts, although differences in muscles, measurement protocol, and reliability time frame preclude a direct between-population comparison.

### 4.2. Associations with Clinical Motor Severity

Myotonometric parameters were not associated with MDS-UPDRS III scores or Hoehn and Yahr stage. This indicates that local resting muscle mechanical properties did not closely reflect global clinical motor severity in the present sample.

This lack of association is plausible because myotonometry and clinical examination assess different phenomena. Myotonometry quantifies the local mechanical response of superficial tissue to an external impulse, whereas clinical rigidity is the resistance perceived during passive joint movement and includes neural, reflex, biomechanical, and examiner-dependent components [2,3]. The MDS-UPDRS III is even broader, combining rigidity with bradykinesia, tremor, gait, posture, and postural stability. Consequently, a strong relationship between the mechanical properties of an individual resting muscle and the total motor score should not necessarily be expected.

Previous studies also suggest that myotonometric properties and clinical rigidity are not interchangeable. Rätsep et al. [20] found that subthalamic stimulation modified both clinical rigidity and resting muscle mechanical properties, but the changes depended on the measurement condition and muscle examined. Similarly, Falletti et al. [9] reported that dopaminergic medication influenced some myotonometric parameters in people with Parkinson’s disease, although the correspondence with clinical motor changes was not uniform. These findings support the interpretation of myotonometry as a complementary local measure rather than a direct instrumental equivalent of clinical rigidity.

The relative homogeneity of our sample may also have attenuated the correlations. Most participants were independently ambulatory, were concentrated in Hoehn and Yahr stages 2–2.5 and had an akinetic-rigid phenotype. Restriction of clinical range reduces between-participant variability and can limit the magnitude of correlations even when an association exists in a broader population.

### 4.3. Associations with Gait Performance

Several moderate nominal associations were observed during preferred-speed walking. Higher tibialis anterior frequency on the more affected side was associated with shorter step length. In the medial gastrocnemius, higher decrement on both sides and longer relaxation time and greater creep on the more affected side were associated with slower preferred gait speed.

The direction of these associations was broadly compatible with altered mechanical behavior of the ankle musculature accompanying poorer habitual gait. The tibialis anterior contributes to ankle dorsiflexion and foot clearance, while the gastrocnemius contributes to stance control and propulsion. Nevertheless, these results should not be interpreted as evidence that resting muscle properties directly caused the gait impairment.

Gait dysfunction in Parkinson’s disease results primarily from impaired movement scaling, reduced automaticity, altered rhythmic control, postural dysfunction, and increased dependence on attentional control [6,21]. Reduced stride length is particularly characteristic of impaired motor automaticity in Parkinson’s disease [21]. Local muscle mechanical properties therefore represent only one possible component of a complex motor disorder.

No nominal associations were observed during maximal-speed walking. None of the gait associations remained significant after FDR correction. The observed coefficients therefore identify possible patterns during preferred-speed walking but do not establish a robust relationship between lower-limb myotonometric parameters and gait performance.

### 4.4. Associations with Manual Dexterity

Higher triceps brachii frequency on the more affected side was nominally associated with longer Grooved Pegboard completion time. The direction of this relationship is compatible with poorer manual performance accompanying higher resting mechanical tone. The Grooved Pegboard Test requires fine manipulation, visuomotor coordination, movement sequencing, and proximal upper-limb stabilization, and its performance may therefore depend on both central motor control and peripheral mechanical conditions.

However, lower triceps decrement was also associated with longer completion time. This finding is less straightforward because lower logarithmic decrement indicates reduced dissipation of the induced oscillation and is commonly interpreted as greater tissue elasticity. The upper-limb findings therefore cannot be reduced to a simple relationship in which greater stiffness consistently corresponds to poorer dexterity. Because this association did not survive FDR correction and involved a proximal muscle measured at rest during a predominantly distal dexterity task, its unexpected direction should be regarded as hypothesis-generating rather than assigned a specific physiological mechanism.

No corresponding associations were found for the biceps brachii, and neither triceps correlation remained significant after FDR correction. However, we must note that in our study, upper-limb myotonometric assessment was limited to the biceps brachii and triceps brachii, which contribute to proximal positioning and elbow stabilization but are only indirect contributors to fine manual dexterity. Forearm and intrinsic hand muscles are more directly involved in Grooved Pegboard performance; however, their myotonometric assessment may be more technically demanding because of their smaller muscle size and the proximity of tendinous, bony, and adjacent muscular structures. Therefore, the present upper-limb findings should be interpreted as associations with proximal muscle properties rather than as a direct characterization of the muscles primarily responsible for fine hand function. Accordingly, these nominal Grooved Pegboard associations should be regarded as indirect and hypothesis-generating rather than as evidence of a direct relationship between the mechanical properties of the muscles primarily responsible for fine hand function and dexterity.

### 4.5. Associations with Functional Mobility

No associations were observed between lower-limb myotonometric parameters and Timed Up and Go performance. The TUG combines standing up, gait initiation, walking, turning, and sitting down and therefore depends on strength, balance, axial mobility, anticipatory postural control, and executive function [13]. Its multidimensional nature may explain why the resting properties of individual muscles showed little relationship with total completion time.

Participants also demonstrated relatively preserved TUG performance, resulting in a restricted range of values. As with clinical severity, this limited variability may have reduced the magnitude of potential correlations.

### 4.6. Interpretation of Nominal and FDR-Corrected Results

Several nominal correlations had moderate magnitudes and broadly plausible directions, but none remained significant following Benjamini–Hochberg correction. The correction was appropriate because the large number of comparisons increased the expected number of false-positive findings [22].

At the same time, the sample was small, several outcomes and myotonometric parameters were related, and the participants were clinically and functionally homogeneous. These factors reduced statistical power and may have limited the detection of moderate associations. Thus, some nominal findings may represent true relationships that the study was insufficiently powered to confirm, but they may also reflect sampling variation.

The a priori sample-size calculation assumed a correlation of r = 0.55, whereas the nominal associations observed in the present study had absolute coefficients of approximately 0.410–0.488. Thus, the study was not powered specifically to detect effects of the magnitude ultimately observed, and the multiple-testing correction further reduced the probability of confirming moderate associations.

The direction of an association can support its biological plausibility but does not establish its validity. This is particularly relevant because not all nominal findings followed a simple “greater stiffness–poorer function” pattern. Reporting both nominal and FDR-corrected results therefore provides the most transparent interpretation: the observed correlations may inform hypotheses, but they should not be considered confirmatory. None of the observed associations should therefore be considered confirmatory.

### 4.7. Limitations

The main limitations were the small and relatively homogeneous sample, which restricted both statistical power and the range of clinical and functional values. The sample was predominantly composed of independently ambulatory participants with an akinetic-rigid phenotype and Hoehn and Yahr stages 2–2.5. Accordingly, the findings should not be assumed to generalize to the broader clinical spectrum of Parkinson’s disease, particularly individuals with substantially milder or more advanced disease or greater mobility impairment. The cross-sectional design also prevents conclusions about causality or temporal direction. In addition, the sample-size calculation was based on the exploratory correlation analyses rather than on an expected ICC or a target precision for the reliability estimates. Thus, some reliability estimates were accompanied by relatively wide confidence intervals, and the precision of the ICCs should be interpreted cautiously. Disease duration was not included as a covariate in the exploratory correlation analyses; therefore, potential confounding by disease chronicity cannot be excluded. The absence of a neurologically healthy comparison group also means that the present study cannot determine whether the observed reliability pattern is specific to Parkinson’s disease or reflects more general properties of MyotonPRO measurement.

All participants were assessed in their usual self-reported ON-medication state. Although this reflects habitual functioning, no standardized clinical confirmation of the ON state was performed, the interval since medication intake was not standardized, and detailed antiparkinsonian medication data were not systematically recorded. These limitations reduce the ability to account for potential variability related to medication type, dose, timing, and individual dopaminergic response.

Measurements were limited to selected superficial muscles at rest and did not characterize deeper tissues, tendons, joint structures, or muscle behavior during active movement. Complete muscular relaxation was not objectively verified using electromyography. Upper-limb myotonometric assessment was limited to the biceps brachii and triceps brachii, which contribute to proximal positioning and elbow stabilization but are only indirect contributors to fine manual dexterity. Forearm and intrinsic hand muscles are more directly involved in Grooved Pegboard performance; however, their myotonometric assessment may be more technically demanding because of their smaller muscle size and the proximity of tendinous, bony, and adjacent muscular structures. Finally, reliability was examined only within the same session and with the same evaluator. The reliability analysis was based on a consistency ICC; therefore, systematic differences between repeated measurement sets were not explicitly incorporated into the reliability estimate. These findings should not be extrapolated to inter-rater or between-session reliability. Although all accepted MyotonPRO sets met the predefined <3% CV quality-control criterion, residual involuntary activity, including tremor or background muscle activation, cannot be completely excluded because relaxation was not objectively verified using electromyography. In addition, the consistency ICC used here quantifies relative within-session reliability but does not establish absolute agreement, systematic bias, standard error of measurement, minimal detectable change, or thresholds for clinically meaningful longitudinal change. Future studies specifically designed for longitudinal monitoring should complement ICC estimates with absolute-agreement and measurement-error indices.

### 4.8. Conclusions

Under the standardized same-session, same-rater protocol used in this study, MyotonPRO measurements of the biceps brachii, triceps brachii, tibialis anterior, and medial gastrocnemius showed good-to-excellent point estimates of within-session reliability, although the precision of some estimates was limited by relatively wide confidence intervals. No associations were found with global clinical motor severity, disease stage, or Timed Up and Go performance.

Several moderate nominal associations were observed with preferred gait and manual dexterity, but none remained significant after FDR correction. These associations are therefore hypothesis-generating only. The relationship between resting muscle mechanical properties and motor performance remains inconclusive, and the present reliability findings should not be generalized beyond the specific same-session, same-rater protocol evaluated here.

## Figures and Tables

**Table 1:** Demographic, clinical, and functional characteristics of the participants.

Variable	Value
Participants, *n*	24
Age, years	66.79 ± 9.15
Sex, men/women	14/10
Disease duration, years	8.34 ± 5.14
MMSE, points	25.30 ± 3.48
MDS-UPDRS III, points	32.96 ± 13.22
Hoehn and Yahr stage 1, *n* (%)	1 (4.2)
Hoehn and Yahr stage 1.5, *n* (%)	4 (16.7)
Hoehn and Yahr stage 2, *n* (%)	9 (37.5)
Hoehn and Yahr stage 2.5, *n* (%)	7 (29.2)
Hoehn and Yahr stage 3, *n* (%)	3 (12.5)
Akinetic-rigid phenotype, *n* (%)	20 (83.3)
Tremor-dominant phenotype, *n* (%)	4 (16.7)
Preferred gait speed, m/s	1.11 ± 0.18
Preferred step length, m	0.69 ± 0.07
Preferred cadence, steps/min	112.79 ± 8.54
Timed Up and Go, s	8.34 ± 2.02
Grooved Pegboard Test, s	159.79 ± 77.08

Values are presented as mean ± standard deviation unless otherwise indicated. Grooved Pegboard performance refers to the clinically more affected hand.

**Table 2:** Intra-rater reliability of the myotonometric parameters.

Muscle	Side	Frequency	Stiffness	Decrement	Relaxation Time	Creep
Biceps brachii	More affected	0.900 (0.770–0.957)	0.849 (0.650–0.935)	0.971 (0.933–0.987)	0.862 (0.682–0.940)	0.880 (0.724–0.948)
Biceps brachii	Less affected	0.868 (0.695–0.943)	0.823 (0.590–0.923)	0.966 (0.920–0.985)	0.829 (0.606–0.926)	0.850 (0.653–0.935)
Triceps brachii	More affected	0.948 (0.879–0.977)	0.940 (0.861–0.974)	0.934 (0.848–0.972)	0.956 (0.897–0.981)	0.961 (0.911–0.983)
Triceps brachii	Less affected	0.898 (0.764–0.956)	0.871 (0.703–0.944)	0.970 (0.931–0.987)	0.938 (0.857–0.973)	0.944 (0.872–0.976)
Tibialis anterior	More affected	0.972 (0.936–0.988)	0.972 (0.936–0.988)	0.976 (0.944–0.990)	0.961 (0.909–0.983)	0.928 (0.834–0.969)
Tibialis anterior	Less affected	0.988 (0.972–0.995)	0.983 (0.961–0.993)	0.988 (0.971–0.995)	0.983 (0.961–0.993)	0.981 (0.956–0.992)
Medial gastrocnemius	More affected	0.956 (0.899–0.981)	0.912 (0.796–0.962)	0.971 (0.933–0.987)	0.939 (0.859–0.974)	0.939 (0.859–0.974)
Medial gastrocnemius	Less affected	0.971 (0.932–0.987)	0.985 (0.965–0.993)	0.971 (0.933–0.988)	0.984 (0.963–0.993)	0.984 (0.963–0.993)

**Table 3:** Associations reaching nominal significance between myotonometric properties and functional outcomes.

Functional Outcome	Muscle	Side	Myotonometric Parameter	r_s_	Unadjusted *p*	FDR-Adjusted *p*
Preferred step length	Tibialis anterior	More affected	Frequency	−0.446	0.029	0.982
Preferred gait speed	Medial gastrocnemius	More affected	Decrement	−0.488	0.016	0.982
Preferred gait speed	Medial gastrocnemius	Less affected	Decrement	−0.410	0.047	0.982
Preferred gait speed	Medial gastrocnemius	More affected	Relaxation time	−0.420	0.041	0.982
Preferred gait speed	Medial gastrocnemius	More affected	Creep	−0.465	0.022	0.982
Grooved Pegboard time	Triceps brachii	More affected	Frequency	0.455	0.026	0.130
Grooved Pegboard time	Triceps brachii	More affected	Decrement	−0.481	0.017	0.130

*r_s_*, Spearman’s rank correlation coefficient; FDR, false discovery rate. Only associations reaching nominal significance (unadjusted *p* < 0.05) are shown. FDR-adjusted *p*-values were calculated using all tests within the corresponding outcome-based family. None remained statistically significant after Benjamini–Hochberg correction; these findings are exploratory and hypothesis-generating. Complete correlation results for maximal-speed walking are reported in Supplementary Table S4.

## Data Availability

The data supporting the findings of this study are available from the corresponding author upon reasonable request, subject to applicable ethical and data-protection restrictions.

## Supplementary Materials

The following supporting information can be downloaded at: https://www.mdpi.com/article/10.3390/s26206410/s1. Supplementary Tables S1–S6 provide the complete correlation matrices for the clinical, gait, manual-dexterity, and functional-mobility analyses; Table S1. Spearman correlations between myotonometric parameters and MDS-UPDRS III scores. Table S2. Spearman correlations between myotonometric parameters and Hoehn and Yahr stage. Table S3. Spearman correlations between tibialis anterior myotonometric parameters and gait outcomes. Table S4. Spearman correlations between medial gastrocnemius myotonometric parameters and gait outcomes. Table S5. Spearman correlations between upper-limb myotonometric parameters on the more affected side and Grooved Pegboard Test completion time. Table S6. Spearman correlations between lower-limb myotonometric parameters and Timed Up and Go completion time. Supplementary Figure S1 provides a global heatmap of the Spearman correlation coefficients across the association analyses. Figure S1. Spearman correlation coefficients between myotonometric parameters and clinical and functional outcomes.
