# Supplementary material for: Reliability of Myotonometric Measurements and Their Associations with Motor Performance in Parkinson’s Disease
Source: Sensors (Basel). 2026 Oct 10;26(20):6410. doi: 10.3390/s26206410 (PMC13709277; doi:10.3390/s26206410)
Supplement: Supplementary file 1 [file sensors-26-06410-s001.zip › sensors-4509610-supplementary.pdf]

Supplementary Materials

Association Between Myotonometric Muscle Properties and Motor Performance in Parkinson's Disease

Table S1. Spearman correlations between myotonometric parameters and MDS-UPDRS III scores.

Parameter	Muscle	Side	r_s
Frequency	Biceps brachii	More affected	0.192
Frequency	Biceps brachii	Less affected	0.232
Frequency	Triceps brachii	More affected	-0.068
Frequency	Triceps brachii	Less affected	0.001
Frequency	Tibialis anterior	More affected	-0.050
Frequency	Tibialis anterior	Less affected	-0.106
Frequency	Medial gastrocnemius	More affected	-0.106
Frequency	Medial gastrocnemius	Less affected	0.097
Stiffness	Biceps brachii	More affected	0.099
Stiffness	Biceps brachii	Less affected	-0.018
Stiffness	Triceps brachii	More affected	0.152
Stiffness	Triceps brachii	Less affected	0.053
Stiffness	Tibialis anterior	More affected	0.073
Stiffness	Tibialis anterior	Less affected	-0.050
Stiffness	Medial gastrocnemius	More affected	0.016
Stiffness	Medial gastrocnemius	Less affected	-0.045
Decrement	Biceps brachii	More affected	-0.238
Decrement	Biceps brachii	Less affected	-0.137
Decrement	Triceps brachii	More affected	-0.009
Decrement	Triceps brachii	Less affected	-0.168
Decrement	Tibialis anterior	More affected	0.228
Decrement	Tibialis anterior	Less affected	0.068
Decrement	Medial gastrocnemius	More affected	0.210
Decrement	Medial gastrocnemius	Less affected	0.284
Relaxation time	Biceps brachii	More affected	-0.177
Relaxation time	Biceps brachii	Less affected	-0.082
Relaxation time	Triceps brachii	More affected	-0.077
Relaxation time	Triceps brachii	Less affected	0.064
Relaxation time	Tibialis anterior	More affected	0.017
Relaxation time	Tibialis anterior	Less affected	0.065
Relaxation time	Medial gastrocnemius	More affected	0.027
Relaxation time	Medial gastrocnemius	Less affected	0.081
Creep	Biceps brachii	More affected	-0.258
Creep	Biceps brachii	Less affected	-0.095
Creep	Triceps brachii	More affected	-0.033
Creep	Triceps brachii	Less affected	0.109
Creep	Tibialis anterior	More affected	0.000
Creep	Tibialis anterior	Less affected	0.057
Creep	Medial gastrocnemius	More affected	0.100
Creep	Medial gastrocnemius	Less affected	0.099

Note: r_s = Spearman's rank correlation coefficient. No correlation was statistically significant at $p < 0.05$.

Table S2. Spearman correlations between myotonometric parameters and Hoehn and Yahr stage.

Parameter	Muscle	Side	r_s
Frequency	Biceps brachii	More affected	0.190
Frequency	Biceps brachii	Less affected	-0.015
Frequency	Triceps brachii	More affected	0.159
Frequency	Triceps brachii	Less affected	0.115
Frequency	Tibialis anterior	More affected	-0.010
Frequency	Tibialis anterior	Less affected	-0.078
Frequency	Medial gastrocnemius	More affected	-0.076
Frequency	Medial gastrocnemius	Less affected	-0.087
Stiffness	Biceps brachii	More affected	-0.127
Stiffness	Biceps brachii	Less affected	-0.281
Stiffness	Triceps brachii	More affected	0.074
Stiffness	Triceps brachii	Less affected	0.213
Stiffness	Tibialis anterior	More affected	-0.025
Stiffness	Tibialis anterior	Less affected	-0.204
Stiffness	Medial gastrocnemius	More affected	-0.133
Stiffness	Medial gastrocnemius	Less affected	-0.004
Decrement	Biceps brachii	More affected	-0.306
Decrement	Biceps brachii	Less affected	-0.339
Decrement	Triceps brachii	More affected	0.192
Decrement	Triceps brachii	Less affected	0.053
Decrement	Tibialis anterior	More affected	0.054
Decrement	Tibialis anterior	Less affected	0.136
Decrement	Medial gastrocnemius	More affected	0.321
Decrement	Medial gastrocnemius	Less affected	0.318
Relaxation time	Biceps brachii	More affected	-0.004
Relaxation time	Biceps brachii	Less affected	0.104
Relaxation time	Triceps brachii	More affected	0.014
Relaxation time	Triceps brachii	Less affected	-0.050
Relaxation time	Tibialis anterior	More affected	0.150
Relaxation time	Tibialis anterior	Less affected	0.149
Relaxation time	Medial gastrocnemius	More affected	0.208
Relaxation time	Medial gastrocnemius	Less affected	0.056
Creep	Biceps brachii	More affected	-0.106
Creep	Biceps brachii	Less affected	0.073
Creep	Triceps brachii	More affected	0.042
Creep	Triceps brachii	Less affected	-0.003
Creep	Tibialis anterior	More affected	0.112
Creep	Tibialis anterior	Less affected	0.123
Creep	Medial gastrocnemius	More affected	0.251
Creep	Medial gastrocnemius	Less affected	0.062

Note: r_s = Spearman's rank correlation coefficient. No correlation was statistically significant at $p < 0.05$.

Table S3. Spearman correlations between tibialis anterior myotonometric parameters and gait outcomes.

Parameter	Gait outcome	Side	r_s	p
Frequency	Preferred speed	More affected	-0.2876	0.173
Frequency	Preferred speed	Less affected	-0.2150	0.313
Frequency	Preferred step length	More affected	-0.4459	0.029
Frequency	Preferred step length	Less affected	-0.2946	0.162
Frequency	Preferred cadence	More affected	-0.1574	0.461
Frequency	Preferred cadence	Less affected	0.0069	0.976
Frequency	Maximal speed	More affected	-0.1559	0.467
Frequency	Maximal speed	Less affected	-0.1637	0.445
Frequency	Maximal step length	More affected	-0.1986	0.352
Frequency	Maximal step length	Less affected	-0.1054	0.624
Frequency	Maximal cadence	More affected	0.0918	0.670
Frequency	Maximal cadence	Less affected	-0.0717	0.739
Stiffness	Preferred speed	More affected	-0.0757	0.725
Stiffness	Preferred speed	Less affected	-0.2098	0.325
Stiffness	Preferred step length	More affected	-0.1691	0.430
Stiffness	Preferred step length	Less affected	-0.3636	0.081
Stiffness	Preferred cadence	More affected	-0.0357	0.869
Stiffness	Preferred cadence	Less affected	0.0183	0.932
Stiffness	Maximal speed	More affected	-0.0296	0.891
Stiffness	Maximal speed	Less affected	-0.1156	0.591
Stiffness	Maximal step length	More affected	-0.1098	0.610
Stiffness	Maximal step length	Less affected	-0.1935	0.365
Stiffness	Maximal cadence	More affected	0.0822	0.703
Stiffness	Maximal cadence	Less affected	-0.0766	0.722
Decrement	Preferred speed	More affected	-0.2437	0.251
Decrement	Preferred speed	Less affected	-0.1736	0.417
Decrement	Preferred step length	More affected	0.0615	0.775
Decrement	Preferred step length	Less affected	0.0497	0.818
Decrement	Preferred cadence	More affected	-0.1022	0.635
Decrement	Preferred cadence	Less affected	-0.0835	0.697
Decrement	Maximal speed	More affected	-0.2038	0.340
Decrement	Maximal speed	Less affected	-0.1419	0.508
Decrement	Maximal step length	More affected	0.1364	0.525
Decrement	Maximal step length	Less affected	0.0736	0.732
Decrement	Maximal cadence	More affected	-0.3136	0.136
Decrement	Maximal cadence	Less affected	-0.3366	0.108
Relaxation time	Preferred speed	More affected	0.0248	0.908
Relaxation time	Preferred speed	Less affected	0.1206	0.575
Relaxation time	Preferred step length	More affected	0.1447	0.500
Relaxation time	Preferred step length	Less affected	0.2092	0.326
Relaxation time	Preferred cadence	More affected	-0.0556	0.796
Relaxation time	Preferred cadence	Less affected	-0.0574	0.790
Relaxation time	Maximal speed	More affected	0.0688	0.749
Relaxation time	Maximal speed	Less affected	0.0089	0.967
Relaxation time	Maximal step length	More affected	0.1555	0.468
Relaxation time	Maximal step length	Less affected	0.0610	0.777
Relaxation time	Maximal cadence	More affected	-0.1431	0.505
Relaxation time	Maximal cadence	Less affected	-0.0452	0.834
Creep	Preferred speed	More affected	0.0100	0.963
Creep	Preferred speed	Less affected	0.0794	0.712
Creep	Preferred step length	More affected	0.1146	0.594
Creep	Preferred step length	Less affected	0.1513	0.480
Creep	Preferred cadence	More affected	-0.0261	0.905
Creep	Preferred cadence	Less affected	-0.0861	0.689
Creep	Maximal speed	More affected	0.0435	0.840
Creep	Maximal speed	Less affected	0.0340	0.875
Creep	Maximal step length	More affected	0.1451	0.499
Creep	Maximal step length	Less affected	0.0858	0.690
Creep	Maximal cadence	More affected	-0.1635	0.445
Creep	Maximal cadence	Less affected	-0.0998	0.643

Note: r_s = Spearman's rank correlation coefficient. Nominal p-values are reported; none remained significant after Benjamini–Hochberg false discovery rate correction.

Table S4. Spearman correlations between medial gastrocnemius myotonometric parameters and gait outcomes.

Parameter	Gait outcome	Side	r_s	p
Frequency	Preferred speed	More affected	0.1771	0.408
Frequency	Preferred speed	Less affected	0.2124	0.319
Frequency	Preferred step length	More affected	0.0331	0.878
Frequency	Preferred step length	Less affected	0.1804	0.399
Frequency	Preferred cadence	More affected	0.0339	0.876
Frequency	Preferred cadence	Less affected	-0.0870	0.685
Frequency	Maximal speed	More affected	-0.0583	0.787
Frequency	Maximal speed	Less affected	0.0984	0.647
Frequency	Maximal step length	More affected	-0.0113	0.958
Frequency	Maximal step length	Less affected	0.1525	0.477
Frequency	Maximal cadence	More affected	-0.0535	0.804
Frequency	Maximal cadence	Less affected	0.0252	0.907
Stiffness	Preferred speed	More affected	0.2559	0.227
Stiffness	Preferred speed	Less affected	0.1754	0.412
Stiffness	Preferred step length	More affected	0.0314	0.884
Stiffness	Preferred step length	Less affected	0.0052	0.981
Stiffness	Preferred cadence	More affected	0.2548	0.229
Stiffness	Preferred cadence	Less affected	0.0600	0.780
Stiffness	Maximal speed	More affected	-0.0422	0.845
Stiffness	Maximal speed	Less affected	0.0605	0.779
Stiffness	Maximal step length	More affected	-0.1394	0.516
Stiffness	Maximal step length	Less affected	0.0170	0.937
Stiffness	Maximal cadence	More affected	0.1235	0.565
Stiffness	Maximal cadence	Less affected	0.0996	0.643
Decrement	Preferred speed	More affected	-0.4878	0.016
Decrement	Preferred speed	Less affected	-0.4095	0.047
Decrement	Preferred step length	More affected	-0.3809	0.066
Decrement	Preferred step length	Less affected	-0.2218	0.298
Decrement	Preferred cadence	More affected	-0.1800	0.398
Decrement	Preferred cadence	Less affected	-0.2478	0.242
Decrement	Maximal speed	More affected	-0.1659	0.439
Decrement	Maximal speed	Less affected	-0.1567	0.465
Decrement	Maximal step length	More affected	-0.0558	0.796
Decrement	Maximal step length	Less affected	0.0205	0.924
Decrement	Maximal cadence	More affected	-0.2422	0.254
Decrement	Maximal cadence	Less affected	-0.2883	0.172
Relaxation time	Preferred speed	More affected	-0.4204	0.041
Relaxation time	Preferred speed	Less affected	-0.2489	0.241
Relaxation time	Preferred step length	More affected	-0.2153	0.312
Relaxation time	Preferred step length	Less affected	-0.0702	0.745
Relaxation time	Preferred cadence	More affected	-0.2243	0.291
Relaxation time	Preferred cadence	Less affected	-0.1009	0.638
Relaxation time	Maximal speed	More affected	-0.0866	0.687
Relaxation time	Maximal speed	Less affected	-0.0936	0.664
Relaxation time	Maximal step length	More affected	-0.0017	0.994
Relaxation time	Maximal step length	Less affected	-0.0392	0.856
Relaxation time	Maximal cadence	More affected	-0.1087	0.613
Relaxation time	Maximal cadence	Less affected	-0.1270	0.554
Creep	Preferred speed	More affected	-0.4648	0.022
Creep	Preferred speed	Less affected	-0.2707	0.201
Creep	Preferred step length	More affected	-0.2863	0.175
Creep	Preferred step length	Less affected	-0.0989	0.646
Creep	Preferred cadence	More affected	-0.2200	0.300
Creep	Preferred cadence	Less affected	-0.1043	0.626
Creep	Maximal speed	More affected	-0.1450	0.499
Creep	Maximal speed	Less affected	-0.1062	0.621
Creep	Maximal step length	More affected	-0.0584	0.786
Creep	Maximal step length	Less affected	-0.0505	0.815
Creep	Maximal cadence	More affected	-0.1296	0.546
Creep	Maximal cadence	Less affected	-0.1187	0.581

Note: r_s = Spearman’s rank correlation coefficient. Nominal p-values are reported; none remained significant after Benjamini–Hochberg false discovery rate correction.

Table S5. Spearman correlations between upper-limb myotonometric parameters on the more affected side and Grooved Pegboard Test completion time.

Parameter	Muscle	r_s	p
Frequency	Biceps brachii	-0.141	0.511
Frequency	Triceps brachii	0.454	0.026
Stiffness	Biceps brachii	0.007	0.974
Stiffness	Triceps brachii	0.076	0.725
Decrement	Biceps brachii	-0.276	0.192
Decrement	Triceps brachii	-0.481	0.017
Relaxation time	Biceps brachii	0.135	0.529
Relaxation time	Triceps brachii	-0.338	0.106
Creep	Biceps brachii	0.049	0.819
Creep	Triceps brachii	-0.351	0.093

Note: r_s = Spearman's rank correlation coefficient. Nominal p-values are reported; none remained significant after Benjamini–Hochberg false discovery rate correction.

Table S6. Spearman correlations between lower-limb myotonometric parameters and Timed Up and Go completion time.

Parameter	Muscle	Side	r_s	p
Frequency	Tibialis anterior	More affected	0.061	0.777
Frequency	Tibialis anterior	Less affected	-0.094	0.661
Frequency	Medial gastrocnemius	More affected	0.050	0.815
Frequency	Medial gastrocnemius	Less affected	0.025	0.908
Stiffness	Tibialis anterior	More affected	-0.105	0.623
Stiffness	Tibialis anterior	Less affected	-0.110	0.610
Stiffness	Medial gastrocnemius	More affected	0.046	0.831
Stiffness	Medial gastrocnemius	Less affected	-0.072	0.737
Decrement	Tibialis anterior	More affected	0.104	0.627
Decrement	Tibialis anterior	Less affected	0.143	0.502
Decrement	Medial gastrocnemius	More affected	0.117	0.583
Decrement	Medial gastrocnemius	Less affected	-0.017	0.940
Relaxation time	Tibialis anterior	More affected	0.083	0.700
Relaxation time	Tibialis anterior	Less affected	0.230	0.280
Relaxation time	Medial gastrocnemius	More affected	0.110	0.606
Relaxation time	Medial gastrocnemius	Less affected	0.112	0.600
Creep	Tibialis anterior	More affected	0.044	0.837
Creep	Tibialis anterior	Less affected	0.179	0.402
Creep	Medial gastrocnemius	More affected	0.166	0.436
Creep	Medial gastrocnemius	Less affected	0.125	0.558

Note: r_s = Spearman’s rank correlation coefficient. No correlation was statistically significant at $p < 0.05$.

Figure S1. Heatmap of Spearman correlation coefficients between myotonometric parameters and clinical and functional outcomes.

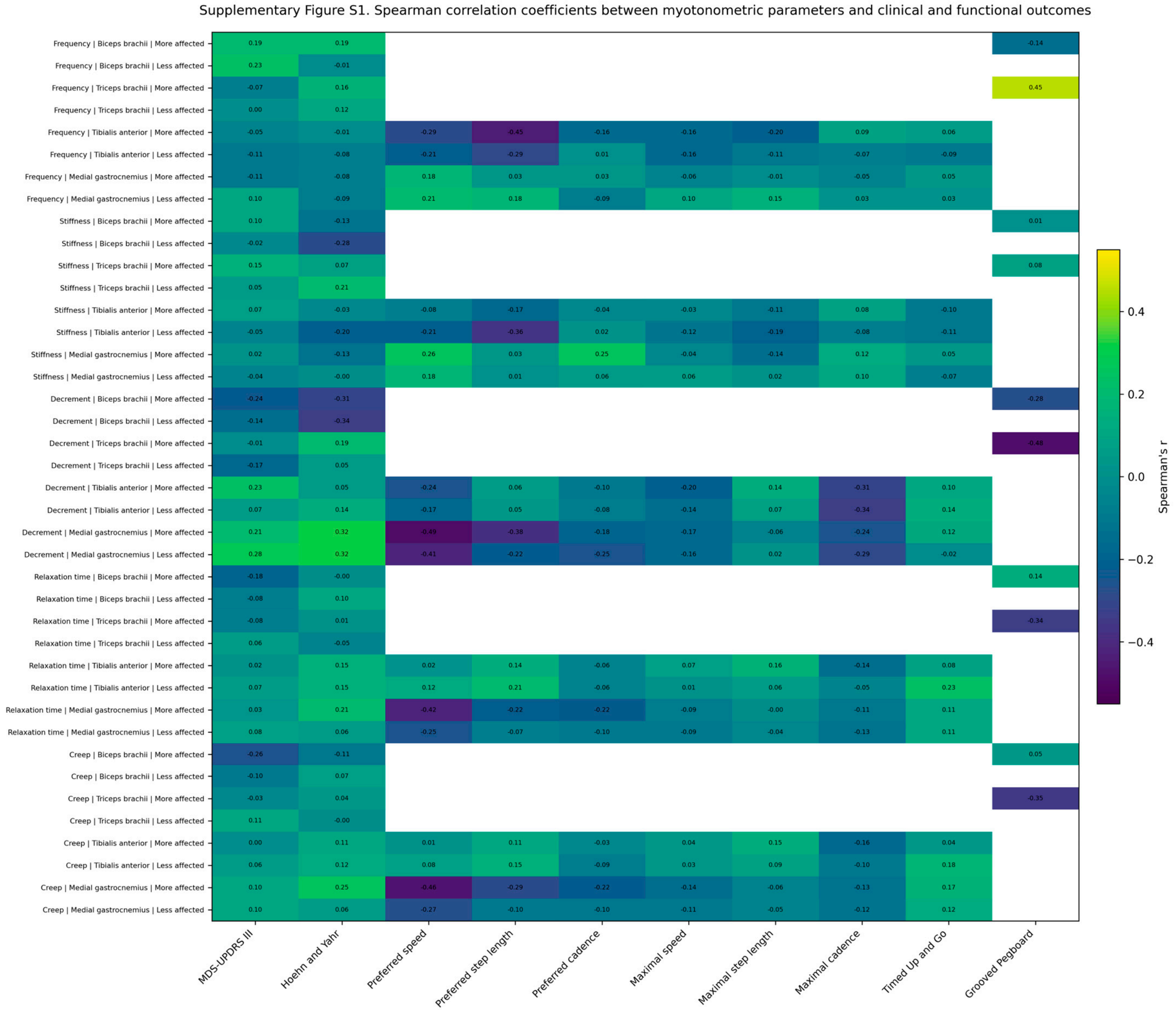

Each cell displays Spearman's rank correlation coefficient. Blank cells indicate combinations that were not analyzed (e.g., Grooved Pegboard correlations for lower-limb muscles). Dual-task gait outcomes were excluded. The figure is intended to display the complete pattern of associations without selectively emphasizing nominally significant findings.
